# COVID-19: Relationship and Impact on Breastfeeding—A Systematic Review

**DOI:** 10.3390/nu13092972

**Published:** 2021-08-26

**Authors:** Marcelino Pérez-Bermejo, Belén Peris-Ochando, María Teresa Murillo-Llorente

**Affiliations:** 1 SONEV Research Group, School of Medicine and Health Sciences, Catholic University of Valencia San Vicente Mártir, C/Quevedo nº 2, 46001 Valencia, Spain; mt.murillo@ucv.es; 2 School of Medicine and Health Sciences, Catholic University of Valencia San Vicente Mártir, C/Quevedo nº 2, 46001 Valencia, Spain; belenperisochando@mail.ucv.es

**Keywords:** COVID-19, SARS-CoV-2, breastmilk, breastfeeding, immune system, vaccine

## Abstract

COVID-19 is an infectious disease caused by the SARS-CoV-2 virus that was declared a Public Health Emergency of International Concern by the World Health Organization (WHO). One major problem faced is whether breastfeeding by mothers infected with the virus is safe. The objective of this work is to study the impact that the SARS-CoV-2 virus can have on breastfeeding, and whether the virus or antibodies can be transmitted from mother to child through milk. We carried out a systematic review of studies focusing on the impact of SARS-CoV-2 on breastfeeding by mothers infected with the virus. The bibliographic search was done through Medline (Pubmed), MedlinePlus and Google Scholar. From 292 records, the title and summary of each were examined according to the criteria, and whether they meet the selection criteria was also analysed. A total of 30 articles are included, of which 26 deal with the study of RNA virus in breastmilk and its involvement in breastfeeding and four on the study of SARS-CoV-2 antibodies in milk. Most studies have been conducted in China. Breastfeeding by mothers infected with SARS-CoV-2 is highly recommended for infants, if the health of the mother and the infant allow for it. Direct breastfeeding and maintaining appropriate protective measures should be encouraged. Should the mother’s health condition not permit direct breastfeeding, infants should be fed with pumped breastmilk or donor milk.

## 1. Introduction

### 1.1. COVID-19 Disease

#### 1.1.1. Epidemiological Description

On 31 December 2019, the Wuhan Municipal Health Commission (Hubei, China) reported 27 cases of pneumonia of unknown aetiology, with a common exposure to a seafood, fish and live animal market in Wuhan, of which seven cases were serious. The onset of symptoms in the first case was on 8 December 2019. On 7 January 2020, a new type of virus from the Coronaviridae family was identified as the aetiological agent of the outbreak, which was subsequently named SARS-CoV-2. On 11 March, the World Health Organization (WHO) declared a global pandemic [1].

Coronaviruses are a family of viruses that cause infection in humans and a variety of animals, including birds and mammals such as camels, cats, and bats. It is a zoonotic disease, which means that it can be transmitted from animals to humans. The coronaviruses that affect humans (HCoV) can produce clinical symptoms that range from the common cold with a seasonal pattern in winter to more severe ones such as those produced by the Severe Acute Respiratory Syndrome (SARS) and Middle East Respiratory Syndrome coronavirus (MERS-CoV) [1]. 

#### 1.1.2. Structure of SARS-CoV-2

SARS-CoV-2 has a spherical morphology with a diameter between 60–140 nm, and 8–12 nm long spikes. Structurally it consists of a nucleocapsid that protects the genetic material (positive-sense single-stranded RNA (+ssRNA) with a length of between 26 and 32 kilobases) and an outer envelope [2]. The SARS-CoV-2 genome encodes four structural proteins: nucleocapsid protein (N-protein), spike protein (S-protein), membrane protein (M-protein) and envelope protein (E-protein). The N-protein, which is phosphorylated, is located in the nucleocapsid and is associated with viral RNA and inserted within the phospholipid bilayer of the outer envelope. The rest of the main proteins are associated with the virus envelope, as well as other accessory proteins such as the hemagglutinin esterase (HE) protein, protein 3 and protein 7a, among others [2]. The S-protein assembles into homotrimers, and forms structures that protrude from the virus envelope. The binding domain to the cell receptor is found in this protein, and is therefore the determining protein of the virus tropism and also the protein that has the fusion activity of the viral membrane with the cell and thus enabling the release of the viral genome within the host cell. The M-protein helps maintain membrane curvature and nucleocapsid attachment and the E-protein plays an important role in the assembly and release of the virus [2,3,4].

#### 1.1.3. Transmission and Pathophysiology

Currently, both the reservoir and the transmitter of the virus to humans are unknown. The most current and widely accepted hypothesis about its origin is that a bat virus evolved towards SARS-CoV-2 through intermediate hosts—suspected to be the pangolin—although the phylogenetic position of the sequence of these viruses is not fully compatible with this hypothesis [1].

COVID-19 spreads mostly from person to person through the inhalation of droplets or fomites from the nose or mouth when an infected person breathes, coughs, sneezes or speaks [5]. These droplets are heavy, so they do not travel far, which is why maintaining a social distance of at least one metre from others is important. Fomites can fall on surfaces and objects and infect other people if they touch them and subsequently touch their eyes, nose or mouth, so maintaining proper hand hygiene is also important [6]. Although vertical transmission is possible, it occurs from mother to child mainly because of their close contact after birth. However, the risk of transmission after delivery is low if protocols from the Spanish Society of Neonatology are followed [7].

Angiotensin-2 converting enzyme (ACE2) is the functional receptor for SARS-CoV-2 since the S-protein recognises ACE2 as its receptor to enter cells. ACE2 is a plasma membrane protein expressed in type I and II alveolar cells, epithelial cells, fibroblasts, endothelial cells, and macrophages [8] and is found primarily in the kidneys, lungs, and heart [9]. ACE2 is responsible for converting Angiotensin I into Angiotensin 1–9 and Angiotensin II into Angiotensin 1–7. Both have anti-inflammatory, vasodilatory, anti-fibrosis effects and also favour natriuresis. All these effects produce a reduction in blood pressure, counter-regulating the action of Angiotensin II. Therefore, ACE2 is associated with protection against arteriosclerosis, hypertension and other vascular and pulmonary processes [10,11]. The binding of SARS-CoV-2 to ACE2 for the internalisation of the virus in the cell can result in preventing it from performing that function [12].

In contrast, the Angiotensin converting enzyme (ACE), which is responsible for transforming Angiotensin I into Angiotensin II, favours the production of secondary peptides with a pro-inflammatory, vasoconstrictor and sodium-retaining effect, which is related to the pathophysiology of arterial hypertension. Severe COVID-19 cases have very high Angiotensin II levels, which have been correlated with the SARS-CoV-2 viral load and lung damage. This imbalance of the renin-angiotensin-aldosterone system (RAAS) could be related to the inhibition of ACE2 by the virus. The same effect had already been observed in the 2003 SARS outbreak [13].

#### 1.1.4. Mechanism of Entry and Innate Immunity

The mechanism of entry into the cell by the virus is through the S-protein that binds to the extracellular domain of ACE2 with high affinity. Cleavage of the S-protein at arginine sites by host protease TMPRSS2 generates S1 and S2 subunits. This step is critical for S2-induced membrane fusion and endocytosis viral internalisation with ACE2 in the lung epithelium. This results in an internalisation of ACE2 by SARS-CoV-2 that results in the loss of the enzyme from the cell surface, preventing a key step from occurring in the degradation of Angiotensin II and generation of Angiotensin 1–7, which, as mentioned above, is a cardiovascular protector. This would exacerbate the damage already done by the virus itself. ACE2 is also highly expressed in the tubular epithelium of the kidney and the loss of this enzyme can contribute to the alteration of sodium transport leading to an increase in blood pressure and both acute and chronic harmful effects in the kidney. Similarly, other organs such as the brain or blood vessels are affected through this enzyme [12]. Neurolipin1 (NRP1) is an important cofactor for virus entry, particularly in cells with low-level ACE2 expression and that it is likely that the high co-expression of ACE2 and TMPRSS2 in nasal epithelial cells favours the high efficiency of SARS-CoV-2 transmission, particularly in two types of goblet cells and a subset of hair cells, which show the highest expression among all cells of the respiratory tree [14]. 

Although the pattern recognition receptors (PRRs) involved in the recognition of SARS-CoV-2 have not yet been accurately determined, the most likely candidates are Toll-like receptors (TLR)3 and TLR7 on the endosome or cytosolic sensors of retinoic acid-inducible gene-1 (RIG-I) and melanoma differentiation-associated gene-5 (MDA5). SARS-CoV-2 infection activates the innate immune system and, after the virus enters the target cell, it is recognised by the TLR3 and TLR7 receptors and the RIG-I and MDA5 viral infection sensors. This recognition induces the type I interferons (IFN) and IFN-stimulated genes response. The TLR3 response triggers the transcription of the NLR family pyrin domain containing 3 gene (NLRP3), which contribute to the activation of the inflammasome NLRP341 and other inflammatory complexes with other cellular responses. The NLRP3 inflammasome induces caspase-1 cleavage and the release of key pro-inflammatory cytokines, interleukin-1ß (IL-1ß) and IL-18, triggering gasdermin D-mediated cell death. Both the degree NLRP3 activation and the release of lactate dehydrogenase (LDH) correlate with the severity of COVID-19 disease. This pathway also makes coagulopathy and thrombotic events common in patients with severe COVID-19 [14,15].

Furthermore, SARS-CoV-2 is capable of inhibiting and delaying the induction of IFN type I by infected cells, leading to a general delay or suppression of IFN type I responses. This allows the virus to replicate and induce more tissue damage, triggering a greater immune response [16]. The ineffective innate immunity of IFN has been strongly associated with a lack of control over the primary infection and a high risk of severe COVID-19, accompanied by innate cellular immunopathology and an increase in plasma cytokines (CXCL10, IL-6 and IL-8) [17,18]. If the delay of the innate immune response is too long—due to particularly efficient evasion by the virus, defective innate immunity, or a combination of both—the virus has an advantage in replicating in the upper respiratory tract and lungs, and the host fails to mount an adaptive immune response fast enough, resulting in conditions that lead to more serious lung disease [19].

#### 1.1.5. Incubation Period

The median incubation period is 5.1 days (95% CI 4.5–4.8). At 11.7 days (95% CI 9.7–14.2), 95% of symptomatic cases have already developed symptoms. Furthermore, the transmission of the infection has been known to begin 2–3 days before the onset of symptoms and up to 7–8 days later in mild cases, but in the most severe cases this transmission would be more intense and lasting.

#### 1.1.6. Clinical Symptoms

Symptoms generally consist of cough, fever, muscle pain, fatigue, sore throat, blocked or runny nose, loss of taste and smell, and shortness of breath or trouble breathing. Diarrhoea, nausea or vomiting, conjunctivitis, and sneezing can be occasional. For this reason, knowing how to distinguish the symptoms from other diseases such as colds, allergies and the flu is essential (Table 1) [5].

In more severe cases, symptoms include breathing problems, persistent chest pain or pressure, confusion, inability to wake up or stay awake, and bluish skin, lips or nails [20]. 

It is estimated that the mean time from the onset of symptoms to recovery is about two weeks when the disease is mild and three to six weeks when it is severe or critical [21]. On the other hand, the time between the onset of symptoms and the possible onset of serious symptoms, such as hypoxaemia, is one week, and between two to eight weeks until death occurs [22].

#### 1.1.7. Adaptive Immunity

It has been widely confirmed that there is a generation of neutralising antibodies during the course of a SARS-CoV-2 infection. A variety of studies have demonstrated that the antibodies with the highest neutralisation power are those that act on a specific area of the S-protein which coincides with the region that binds to human cells known as RBD (Receptor Binding Domain). The tests that have been carried out found that this RBD can undergo mutations, which means including a miscellany of neutralising antibodies that target the various areas of S-protein in therapeutic and vaccine creation strategies is essential [23,24,25]. To understand this, we must understand adaptive immunity. The adaptive immune system comprises three main cell types for viral infections: B cells that produce antibodies, CD4^+^ T cells that possess a range of helper and effector functionalities, and CD8^+^ T cells that kill infected cells. This type of immune response is slow because of the intrinsic need to select and expand specific cells against the virus from large groups of naive B cells and T cells specific for different molecular structures and sequences (more than 10^9^ cells each). This proliferation and differentiation of naive cells in effector cells requires approximately 6–10 days for them to take control of the viral infection [19].

SARS-CoV-2, is highly effective in delaying the activation of innate intracellular immune responses so the virus initially replicates unabated until innate immune alarms occur. This delay in innate immune responses is sufficient to produce an asymptomatic infection [26] or a clinically mild disease. The presence of T cells and antibodies is associated with the successful resolution of an average COVID-19 case [27]. In studies of COVID-19 patients, SARS-CoV-2 specific T cell responses have been found to be significantly associated with milder disease [19].

T cell responses are detected after almost all SARS-CoV-2 infections [27,28]. Within this cell type, CD4^+^ T cells produce a greater response than CD8^+^ T cells [27] and have been associated with the control of primary infection [28]. The prevalence and magnitude of CD4^+^ T cell responses correlate with the level of expression of each SARS-CoV-2 protein with the main targets being spike protein, M-protein and nucleocapsids [27]. Recognised SARS-CoV-2 CD4 CD4^+^ T cell antigen patterns appear to be similar during acute infection, convalescence and memory phases [27,28], although ORF7/8 CD4^+^ T cell responses may exhibit relative selectivity for the acute phase [19].

SARS-CoV-2-specific CD4^+^ T cells can be detected as early as 2–4 days after the onset of symptoms. Notably, these cells had the strongest association with a decreased severity of COVID-19, compared to antibodies and CD8^+^ T cells. Rapid induction of CD4^+^ T cells in acute-phase COVID-19 was associated with mild disease and accelerated viral clearance. By contrast, the markedly widespread absence of these cells was associated with severe COVID-19 [19,28]. SARS-CoV-2-specific CD4^+^ T cells commonly differentiate into Th1 cells and follicular T helper cells (Tfh) [29]. Circulating SARS-CoV-2-specific Tfh cells (cTfh) are generated during acute infection, as are memory cTfh cells for SARS-CoV-2. Although neutralising antibody titers have not been correlated with reduced disease severity, virus cTfh cell frequencies have been associated with reduced severity [19]. Notably, a substantial fraction of SARS-CoV-2 cTfh is CCR6^+^, potentially indicative of mucous airway conduction, which has also been observed in the HKU1 common cold coronavirus [30]. Although we are still unsure of which exact CD4^+^ T cells provide this CD8^+^ help, we suspect that IL-21 may play an important role, which is a cytokine of the aforementioned Tfh cell [27,28]. 

The vast majority of individuals infected with the virus seroconvert within 5–15 days after the onset of symptoms, with 90% seroconversion on day 10 [28]. The primary antigens examined for seroconversion are the S and N-proteins. Immunoglobulin G (IgG) titres of the S and N-proteins are highly correlated. The target of neutralising antibodies is the spike protein and the receptor-binding domain (RBD) of this protein is the target of more than 90% of those antibodies, while a small proportion target the N-terminal domain. Estimates of seroconversion to SARS-CoV-2 spike protein range from 91–99% in large studies. Spike protein IgG, IgA and IgM develop simultaneously in infected individuals [19].

In general, and in various animal models, higher antigen load drives higher antibody titres. This appears to be true in the case of SARS-CoV-2, where neutralising antibody titres—and total spike protein antibody titres—have been positively correlated with disease severity in large cohort studies [19,31]. 

#### 1.1.8. Interpersonal Variation of Immunity 

The human immune system is inherently diverse from person to person and there is no scenario where 100% of people respond to a viral infection in an identical manner. Pre-existing immunity may also contribute to the heterogeneity of COVID-19 in the population [19].

Age is the greatest risk factor for the development of severe or fatal COVID-19 because elderly individuals generally produce a worse coordinated adaptive immune response to SARS-CoV-2. On the other hand, paediatric SARS-CoV-2 infections are currently not fully understood. Children under the age of 13 generally have mild or no symptoms. Children generate different antibody responses to SARS-CoV-2 than adults do. In rare cases, infected children develop MIS-C syndrome, which appears to be an autoimmune condition that develops after infection, conceptually similar to Kawasaki’s disease [19].

Regarding sex, males are at somewhat higher risk than females for severe COVID-19. In particular, 10% of severe cases of this disease are in people with type I IFN autoantibodies, and more than 90% of those cases were male [19]. However, several studies found that males had higher spike protein IgG and RBD and nucleocapsid IgG. In contrast, no differences were observed in IgA or PSV neutralising titres, nor in the frequency of memory B cells or memory CD4^+^ and CD8^+^ T cells between males and females [32]. 

Furthermore, the IgG titres of the spike protein and RBD were higher in hospitalised cases than in non-hospitalised ones. The frequencies of spike and RBD specific memory B cells were also higher in hospitalised cases (approximately 1.7 times and 2.5 times, respectively). In contrast, the frequency of memory CD8^+^ T cells was not higher in hospitalised patients, and the frequency of memory CD4^+^ T cells was lower in hospitalised cases compared to those who had not been hospitalised. This suggests that long-term humoral immunity is greater in individuals experiencing a more severe disease course. Memory T cells did not follow the same pattern, consistent with indications that hospitalised COVID-19 cases may be associated with poorer T cell responses in the acute phase. Furthermore, these data show that, while sex and severity of COVID-19 contribute to differences in immune memory, none of the factors could explain most of the heterogeneity in the immune memory of this virus [32].

#### 1.1.9. Duration of the Immunity

A certain study [33] saw T cell responses occurring six months after infection in relatively mild or asymptomatic infections. However, the magnitude of responses was highly variable within the cohort, and a correlation of this response was the presence of symptoms early in the infection. People with symptomatic infections had significantly higher IFNγ-producing T cell responses at six months after infection compared to people with asymptomatic infection. The greater response of CD4^+^ T lymphocytes compared to CD8^+^ lymphocytes is consistent with the findings of another report [32] that analysed the response six to eight months after infection. Interestingly, IL-2, with or without IFNγ, was the dominant CD4 cytokine produced in response to both stimulation by the S-protein of the virus as well as the other proteins. 

The exact durability of immunity is unknown at this time, however. In a study carried out on a series of cases, it was found that there was a reduction in the neutralising capacity of antibodies in the two to three months following exposure, known as the early convalescence period, in 11.7% of asymptomatic cases and 8.3% of mild symptomatic cases. These data suggest that a weaker immunity is generated in asymptomatic cases compared to cases in which symptoms appear. However, the significance of researching the loss of protective immunity over time is still doubtful [34].

#### 1.1.10. Severity of the Disease

The severity of the disease caused by SARS-CoV-2 increases with other comorbidities. Chronic kidney disease, cardiovascular disease, arterial hypertension and diabetes mellitus are among the comorbidities that have the highest risk of triggering serious symptoms. These are followed by immunodeficiencies, smoking, chronic respiratory disease and chronic liver disease [35].

#### 1.1.11. Mortality Rate

“Death from COVID-19” has been defined as death resulting from a clinically compatible disease in a probable or confirmed case of COVID-19, unless there is a clear alternative cause of death that cannot be related to that disease [36]. A study carried out in the United States comparing mortality from COVID-19 (March–October 2020) with the main causes of mortality prior to the pandemic (March–October 2018) showed that COVID-19 was the leading cause of death at certain times. Compared to the leading causes of death during the same period in 2018, COVID-19 was the third leading cause of death in children and adults (697.5 deaths/million), ranking only behind heart disease (1287.7 deaths/million) and cancer (1219.8 deaths/million). However, these figures probably underestimate the true excess mortality by at least 20%, due, in part, to the indirect effects of the pandemic on non-COVID-19 diseases. This article also highlighted that, in autumn 2020, starting on 1 November and reaching its maximum peak on 9 December (3411.1 deaths/million), COVID-19 became the leading cause of death [37]. However, the risk of death is higher in older populations and lower in young people. Compared with individuals aged 18–29, individuals aged 75–84 and those older than 85 have a 200- and 630-fold increased risk of death, respectively [38]. In addition, residents of nursing homes and long-term care centres are at high risk as seen by the data; they represent only 5% of the population but 33% of mortality [39]. Globally, according to the World Health Organization, total accumulated deaths stand at 38,705 per 100,000 people [40].

#### 1.1.12. COVID-19 Vaccines

There are several types of COVID-19 vaccines, but the most relevant to this study because they are more widely applied to pregnant women are mRNA (messenger RNA). This type of vaccine is composed of 5′ capped single-stranded capped mRNA which encodes the SARS-CoV-2 S-protein. mRNA is produced by in vitro transcription from a corresponding DNA model, in a cell-free medium. Depending on the type of vaccine, each 0.3 mL dose contains 30 µg of this highly purified mRNA embedded in lipid nanoparticles (Pfizer-BioNTech) and the other 0.5 mL dose contains 100 µg (Moderna) [41,42]. 

The vaccines’ mechanism of action is that the formulation of the mRNA in lipid nanoparticles allows it to enter the host cells (in the case of Moderna, mainly in the dendritic cells and macrophages of the subcapsular sinus) without degrading it. The expression of genetic information by the cellular machinery produces the SARS-CoV-2 S-protein, which occurs on the surface of the cell. Detection of this antigen induces an immune response against the S-protein, both in neutralising antibodies and in cellular immunity, which is the basis of protection against COVID-19. As it does not contain live viruses or the complete genome, the vaccine has no replicative capacity and cannot cause disease. As mRNA is processed directly in the cytoplasm, it cannot be integrated into the host genome. mRNA is usually naturally degraded in approximately 48 h [41,42]. 

In terms of efficacy against COVID-19, they differ slightly. For Pfizer-BioNTech, the primary efficacy analysis of the phase 3 study included 36,621 participants aged 12 or older (18,242 in the COMIRNATY vaccine group and 18,379 in the placebo group), with no evidence of previous SARS-CoV-2 infection up to seven days after the second dose. The results for participants aged over 16 showed that eight confirmed cases of COVID-19 were found in the vaccinated group and 162 cases in the placebo group seven days after the second dose. Efficacy in participants without evidence of prior SARS-CoV-2 infection was 95% (95% CI: 90.0–97.9%); in the group aged 65 years or older, the efficacy was 94.7% (95% CI: 66.7–99.9%) and in those aged 75 years or over it was 100%, but with a non-significant confidence interval (95% CI: −13.1–100%). Efficacy in participants with or without evidence of prior SARS-CoV-2 infection was 94.6% (95% CI: 89.9–97.3%). With the available data, optimal protection cannot be ensured until seven days after receiving the second dose [42].

Regarding the efficacy of the Moderna vaccine, the primary analysis of the phase 3 study included 28,207 participants aged 18 or older (14,134 in the vaccine group and 14,073 in the placebo group), with no evidence of previous SARS-CoV-2 infection up to 14 days after the second dose. The results for participants aged over 18 showed that eight confirmed cases of COVID-19 were found in the vaccinated group and 185 cases in the placebo group 14 days after the second dose. Efficacy in participants without evidence of prior SARS-CoV-2 infection was 94.1% (95% CI: 89.3–96.8%); in the group aged 65 years or older, the efficacy was 86.4% (95% CI: 61.4–95.2%) and in the 18 to 65 group, 95.6% (95% CI: 90.6–97.9%). Efficacy in participants at high risk of severe COVID-19 infection was 94.4% (95% CI: 76.9–98.7%). With the available data, optimal protection cannot be ensured until 14 days after receiving the second dose [41].

### 1.2. Pregnancy and COVID-19

There is currently no scientific evidence of a greater susceptibility to SARS-CoV-2 infection in pregnant women compared to those who are not [21]. However, at the start of the pandemic it was thought that the clinical characteristics of pregnant women with COVID-19 were similar to those women who were not pregnant and it was also thought that they not more predisposed to developing severe pneumonia or death [43]. 

A systematic review analysing 42 studies that included 438,548 pregnant women found that compared to the absence of SARS-CoV-2 infection in pregnancy, COVID-19 was associated with pre-eclampsia, preterm delivery, and natal mortality. Compared to mild COVID-19, severe COVID-19 was strongly associated with pre-eclampsia, preterm labour, gestational diabetes, and low birth weight. Pregnant women and babies can be particularly susceptible to this disease because the physiological changes of pregnancy involve the cardiorespiratory and immune systems, which can result in an altered response to infection. Foetuses can be exposed to the virus during critical periods of foetal development. Several studies have found that pregnant women are not at increased risk of suffering from the disease but their risk of severe COVID-19 may be increased and there may be an increased risk of complications in the foetus and in pregnancy [44]. 

#### Vaccine against COVID-19 in Pregnant Women

Although the clinical trials of the vaccines against COVID-19 did not include pregnant women, the available data, mainly on the use of the vaccines in the United States, did not find any adverse effects on pregnancy. A recently published study in the US that included 36,591 pregnant women who had been vaccinated against COVID-19 with mRNA vaccines, found no safety signals [42]. 

The UK Joint Committee on Vaccination and Immunisation (JCVI) recommends offering COVID-19 vaccines to pregnant women at the same time as the rest of the population, according to their age and clinical risk. Given that the Comirnaty and Moderna vaccines have had larger trials that include pregnant women, they are recommend for use; although for those who received a first dose of Vaxzevria, completing the regimen with the same vaccine in that country (United Kingdom) was recommended [42]. 

Based on the available data—which have found no adverse effects on pregnancy—and the recommendations of other countries, vaccinating pregnant or lactating women with mRNA vaccines when appropriate is proposed, according to the prioritisation group they are in [42]. A prospective cohort study of 131 women of reproductive age who received the vaccine (84 pregnant, 31 lactating and 16 non-pregnant women) found that mRNA vaccines against COVID-19 generated strong humoral immunity in pregnant and lactating women, with immunogenicity and reactogenicity similar to those observed in non-pregnant women. Vaccine-induced immune responses were statistically significantly greater than the natural response to infection [45].

### 1.3. Breastfeeding

As soon as a baby is born, it requires close contact with its mother for nurture and to breastfeed, and the breastmilk they drink plays a significant role in the child’s present and future health. A large number of human studies on early maternal separation from nursing babies (or preterm nursing babies) focus primarily on the first 1–2 h (or first days after delivery) of skin-skin interaction of nursing babies and their mother and its possible effects on breastfeeding, cortisol levels, crying, sleep, pain reduction, and the physiological, emotional and cognitive regulation of the infant, etc. [46].

Breastfeeding has been shown to be a protective factor against various infectious, atopic diseases, cardiovascular diseases, leukaemia, necrotising enterocolitis, coeliac disease and inflammatory bowel disease. Similarly, it has a positive impact on neurodevelopment, improving IQ and may reduce the risk of other conditions such as attention deficit, generalised developmental disorders and behavioural disorders. Breastfeeding can prevent 13% of infant mortality worldwide, and reduces the risk of sudden infant death by 36%. This type of breastfeeding also implies direct savings from a reduced use of breastmilk formulas, and indirect savings in associated health costs, premature deaths and quality-adjusted life years, among others. In addition, it is environmentally beneficial since it does not leave a carbon footprint in its production and consumption. The use of breastmilk formulas has associated inherent risks, increases the risk of disorders of the oral cavity, such as mouth breathing, malocclusion, alteration of the bite and cavities. Finally, the intestinal microbiota, oxygenation and thermoregulation of nursing babies are negatively affected by the use of these breastmilk formulas. This is known as the imprinting of motherhood in the neural system: the newborn’s first contact and relationship with their environment stabilises its respiration, temperature and heart rate parameters [21,47].

This is why breastfeeding has a positive impact on infant morbidity and mortality. Furthermore, both maternal and donated breastmilk can reduce the risk of different pathologies since it is rich in antibodies that provide the newborn with their first source of adaptive immunity. In addition, it contributes to a complete, balanced, sufficient and adequate diet by not requiring preparation and always at the right temperature [47,48]. 

Breastfeeding should be started in the first hours after delivery as it has, as mentioned above, a multitude of protective properties. Likewise, it provides the necessary nutrients for the healthy development of the baby. For the mother it reduces the risk of postpartum depression, anaemia and different pathologies [21].

Respiratory infections are one of the leading causes of morbidity in children. During the first year of the baby’s life, breastfeeding will protect them against these infections, depending on its duration, mainly against those of the lower respiratory tract [49]. The immaturity of the baby’s immune system at birth increases the risk of infection by external agents, including viruses and bacteria; the underdevelopment of the neonatal respiratory and gastrointestinal tracts makes it difficult to resist invasion [50].

#### Composition of Breastmilk

Breastmilk contains carbohydrates, proteins, fats, vitamins, minerals, digestive enzymes, and hormones and is rich in immune cells, including macrophages, stem cells, and many other bioactive molecules. Some of these molecules are derived from proteins and lipids, while others are derived from indigestible proteins, such as oligosaccharides. Human breastmilk oligosaccharides (HMO) contain anti-infective properties that fight pathogens in the infant’s gastrointestinal tract, such as salmonella, listeria and campylobacter. In addition, they also play a vital role in the development of a diverse and balanced microbiota, essential for appropriate innate and adaptive immune responses, and help colonise up to 90% of the infant biome [48,51].

Human breastmilk is a complex matrix with an overall composition of 87% water, 3.8% fat, 1% protein, and 7% lactose. Fat and lactose, respectively, provide 50% and 40% of the total energy in breastmilk. During early lactation, the protein content in breastmilk ranges from 1.4–1.6 g/100 mL, to 0.8–1.0 g/100 mL after three to four months, and to 0.7–0.8 g/100 mL after six months. Fat content varies significantly with the maternal diet and is also positively related to weight gain during pregnancy. It has been observed that breastmilk is almost always adequate in essential nutrients for the growth and development of full-term babies, even when the mother’s nutrition is inadequate [48].

Despite the work carried out to date, there is still much uncertainty in daily clinical practice regarding the safety of nursing babies and the perceived advantages and disadvantages of interrupting breastfeeding by SARS-CoV-2–infected mothers [50]. This review was carried out because of all the above. Our objectives were to analyse the impact of SARS-CoV-2 on breastfeeding, study the proper management of breastfeeding by mothers infected with SARS-CoV-2 and analyse the latest evidence on the effect of new vaccines on breastfeeding and recommendations.

## 2. Search Methodology

**Figure 1:**
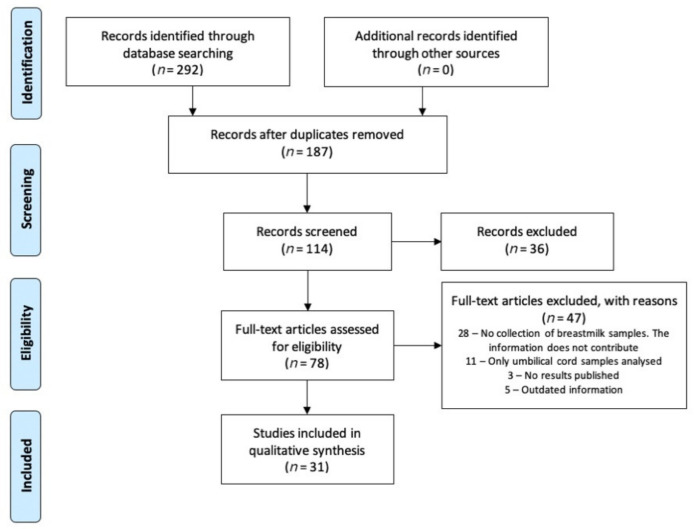
PRISMA flowchart of study selection process.

This systematic review was conducted in accordance with the criteria set forth in the Preferred Reporting Items for Systematic Reviews and Meta-Analysis (PRISMA) [52]. The literature search was carried out in PubMed and Web of Science. The search strategy combined the MeSH terms “COVID-19”, “SARS-CoV-2”, “breastfeeding”, “immune system”, “breastmilk”, and “vaccine”, combined with each other using Boolean operators. Articles less than 2 years old were selected (COVID-19 period), obtaining a total of 31 articles to review. The flowchart in Figure 1 details the screening and selection process. All articles were about COVID-19 and its possible impact on breastfeeding. 

As the studies admitted for review used a variety of methodologies including case reports, case series, and cross-sectional studies, we decided to use relevant checklists from the Joanna Briggs Institute (JBI) [53], to assess study design and quality. One point was ascribed to each criterion achieved on the checklist. The quality of the studies was rated as a percentage of the total available points on each checklist. 

## 3. Results

As shown in Figure 1, the literature search identified 292 records. After removing duplicates and screening titles and abstracts, 114 articles were selected for full-text review, of which 31 studies met the inclusion criteria [45,54,55,56,57,58,59,60,61,62,63,64,65,66,67,68,69,70,71,72,73,74,75,76,77,78,79,80,81,82,83]. The articles were divided into two groups for review and analysis. The first group included studies on the detection of virus RNA in breastmilk and its impact on breastfeeding (Table 2), and the second, studies on the detection of SARS-CoV-2 antibodies in breastmilk (Table 3). Table 4, Table 5 and Table 6 show the quality assessment of the studies.

## 4. Discussion

Increasingly more studies are available on the impact of the SARS-CoV-2 virus on breastfeeding. Understanding the positive and negative impacts it can have is essential if we want to respond appropriately.

The colostrum from mothers who have tested positive to SARS-CoV-2 has been analysed using molecular diagnostic techniques by real-time PCR, and SARS-CoV-2 RNA was not detected in any of the samples obtained in the first few hours following delivery, after the neonate has had their first feed. Additionally, none of the neonates developed COVID-19 symptoms nor did they test positive for it [54,55,56,57]. Furthermore, it has been seen that angiotensin II converting enzyme receptors have a very low expression in the placenta, which makes the chances of vertical transmission through the placenta very low [55], so the most frequent form of transmission to the neonate is through respiratory secretions [57]. This low probability of transmission from mother to neonate through breastmilk concurs with other studies [64,69,74] where the breastmilk samples also did not contain SARS-CoV-2 RNA and highlight the fact that the risk of transmission is very low.

However, in another study [58], SARS-CoV-2 was identified in breastmilk obtained more than a week after giving birth, although the sample was collected using an electric pump. This study analysed the breastmilk of two SARS-CoV-2 infected lactating mothers. After admission and delivery (day 0), four samples from Mother 1 were negative, but SARS-CoV-2 RNA was detected in Mother 2’s breastmilk on days 10, 12 and 13, although later samples were negative. The detection of viral RNA in Mother 2’s breastmilk coincided with the onset of mild COVID-19 symptoms and a positive SARS-CoV-2 diagnostic test for her newborn. However, whether this newborn was infected through breastfeeding or another form of transmission was unclear.

Another study [59] analysed five pregnant women who were hospitalised and clinically diagnosed with COVID-19. During follow-up, three of the four available serum samples had significantly elevated levels of SARS-CoV-2 IgM and IgG. Importantly, four of the five (80%) breastmilk samples were negative for SARS-CoV-2 RT-PCR, which is similar to the previous observations, while one (20%) patient (Patient 3) showed SARS-CoV-2 RNA in her breastmilk. Additionally, the breastmilk samples from Patient 3 after delivery—during days two and three—remained positive for SARS-CoV-2. It should be noted that the Ct value of the RT-PCR test was relatively high (38.2 and 38.5), suggesting the presence of SARS-CoV-2 in the breastmilk of a patient with COVID-19 persists.

Results from previous studies that tested human breastmilk for the presence of SARS-CoV-2 suggested that breastmilk may act as a potential vehicle for mother-to-child transmission. However, no viable virus has been detected in breastmilk, only RNA, and contact transmission could not be ruled out when RNA had been detected. Most previous studies are limited in that samples contained few participants, were cross-sectional, and/or did not report on how the breastmilk was collected and/or analysed. Although the detection of SARS-CoV-2 RNA in breastmilk and/or breast is worrisome, it does not necessarily indicate the presence of viable or infectious viruses. In the only study that evaluated the viability of SARS-CoV-2 in breastmilk, a single breastmilk sample that tested positive for SARS-CoV-2 RNA did not contain replication-capable viruses [1,69,81]. 

Many studies concur that both direct and pumped breastfeeding should be encouraged when the mother is infected but the neonate is not, while taking appropriate precautionary measures, to avoid contagion through respiratory secretions, when the mother’s disease is mild or moderate and there is a possibility of transmitting it [50,54,55,56,64,68,72,73,74,75,76,77,78]. According to these studies, breastfeeding can improve the health of both the mother and the neonate, so the mother should not be separated from her infant when the disease is mild. The percentage of neonates who were infected was very low and when there were infections, it could not be shown that the cause was breastmilk [74,75,76]. Furthermore, certain studies not only encouraged breastfeeding, but also argued that breastfeeding played an important protective role against the disease, given that there was a possible induction of passive immunity against SARS-CoV-2 [64,72,74]. In some cases [73,76,79], breastfeeding is encouraged even when breastmilk samples contain virus RNA since, as mentioned above, virus RNA does not indicate that the virus is viable.

Despite not finding virus RNA in breastmilk samples, certain studies encourage infected mothers to breastfeed their uninfected children with precautionary measures, but only after maternal isolation (they recommend breastfeeding while the mother is isolated), the nasopharyngeal PCR result is negative, or SARS-CoV-2 RNA is not found in the breastmilk [61,62,66]. Conversely, other studies, although there are fewer of them, recommend temporarily stopping breastfeeding when the mother is infected [65,73] regardless of whether the breastmilk sample contains virus RNA and, if it does, they recommend the decision to continue breastfeeding be made by the parents and the doctor [63].

When both the mother and the infant are infected with COVID-19, regardless of whether the breastmilk sample contains SARS-CoV-2 RNA, studies recommended breastfeeding newborns either directly or with pumped breastmilk, since breastmilk provides many benefits for both mother and neonate [69,70,71]. Other studies emphasised that repeated breastmilk samples collected from women after testing positive for COVID-19 did not contain SARS-CoV-2 RNA; however, the risk of transmission through the skin of the breast should be further evaluated. It is important to note that breastmilk produced by infected mothers is a source of anti-SARS-CoV-2 IgA and IgG and has the ability to neutralise SARS-CoV-2 activity. This may indicate possible immune protection for neonates. On this basis, these results support recommendations that mothers with mild to moderate COVID-19 should continue breastfeeding [81,82]. One study analysed the immunological characteristics and the evolution of the disease of four mother-baby pairs and found similar results. Three breastmilk samples tested positive for SARS-CoV-2 IgM or IgG. Three neonates tested positive for SARS-CoV-2 IgG and one tested positive for IgM within 24 h of birth. Because a low risk of transmission of the virus was seen, this study, as in many others, recommends mothers continue breastfeeding with appropriate precautions since babies can benefit from the direct acquisition of antibodies against the SARS-CoV-2 virus [80].

In other studies of mothers who received SARS-CoV-2 mRNA vaccines [45,83], a robust secretion of IgA, IgM and IgG antibodies against the virus was found in breastmilk for six weeks after vaccination. In one of them, the three types of antibodies (IgA, IgM and IgG) were found in all samples and IgA and IgG antibodies were found in 86.1% and 97% of the samples, respectively. Furthermore, in both studies, an immune transfer to neonates through breastmilk was observed and these antibodies showed neutralising effects against SARS-CoV-2, which may indicate a potential protective effect against infection in the infant.

Currently, the role of antibodies found in breast milk from COVID-19 infected mothers on infants has not been specifically studied. In most studies analysing breast milk, reference is made to the fact that future researchers should incorporate not only the presence of SARS-CoV-2 specific antibodies in human milk, but also the maturation, affinity, and functionality of these antibodies [84,85]. IgA antibodies were detected in a study that tested the milk of 2 infected mothers. While this study was not designed to evaluate the level of protection that IgA conferred from mother to infant during prolonged lactation periods, patterns of protection found in other respiratory viruses suggest this confers effective passive immunity, but protection against SARS-CoV-2 infection specifically has yet to be established [85]. 

This limitation is present not only in studies of mothers who have passed the infection, but also in those in which the breast milk of mothers vaccinated against SARS-CoV-2 has been analysed, and it is also mentioned that additional studies are needed to evaluate the effect of these vaccines on lactation outcomes and infant health because the protection they may provide to the child has not been studied [82,86,87]. 

In another article, after de vaccination of 10 mothers, the transfer of vaccine-induced IgG antibodies to the newborn was demonstrated. However, there is also no mention of the effect of these antibodies on the infant and, as in most articles, it only focuses on antibody titres rather than T cell-driven or other functional immunity [45]. 

This limitation also occurs in more recent studies where they stress the importance of demonstrating the neutralizing ability of the antibodies and the duration of antibody response in human milk, which will allow better clinical management of this issue [88]. In addition, in the studies in which has been demonstrated the ability of the antibodies to neutralize the virus, it has been done in vitro. So that it has not been possible to study the impact that these antibodies may have on the infant and more studies are required to determine if these antibodies confer passive immunity to breastfed infants [80,89].

The WHO, the United Nations International Children’s Emergency Fund (UNICEF), the Union of European Neonatal and Perinatal Societies (UENPS) and the US Centers for Disease Control and Prevention (CDC), have all unequivocally encouraged breastfeeding during the pandemic, emphasising the long-term immunological and psychosomatic benefits of breastfeeding. Current recommendations indicate that there is insufficient evidence showing that COVID-19 is transmitted through breastmilk. On this basis, strict separation measures between mother and child and interruption of breastfeeding should be avoided, regardless of a positive diagnosis and symptoms, unless the severity is such that the mother cannot care for her child, in which case the baby should be given fresh, unpasteurised breastmilk [50]. Measures taken to minimise the risk of transmission during breastfeeding include the use of a mask, frequent washing of hands, cleaning and sterilisation of infant feeding equipment before and after use, washing the breasts with soap and water and avoiding falling asleep with the baby. Should the above not be possible, pumping the breastmilk and having the baby fed by a healthy family member or caregiver is also recommended [50,54,83].

The main limitation of this work is that many of the studies found are descriptions of a single case, not having found works with a more adequate sample size. Two great unknowns still remain to be further researched. One of them is whether the SARS-CoV-2 RNA found in breastmilk is a viable virus or not, since there are few studies on this issue, although the few that have been carried out indicate that it is not. The other unknown is how long the antibodies that are transferred through breastmilk to the neonate last. 

## 5. Conclusions

Various scientific studies have found that there is the presence of antibodies against SARS-CoV-2 in breastmilk, both when the mother contracts the disease and after the vaccine against the virus has been administered. Although there is a transmission of antibodies against SARS-CoV-2 through breastmilk, it does not appear to be contagion of the disease; SARS-CoV-2 RNA has been found in breastmilk, but no viable viruses have. Thus, infants can benefit from the direct acquisition of antibodies against SARS-CoV-2 through breastmilk. Results from multiple studies support recommendations to continue breastfeeding during mild to moderate maternal COVID-19 illness, as breastmilk likely provides specific immune benefits to infants. Despite the pandemic, international organisations continue to recommend breastfeeding since it is safe with mild-moderate infection and when appropriate control measures are taken to reduce the risk of contagion (person-person) through respiratory secretions between the mother and the baby. When mother–baby separation occurs because the mother is isolated, the neonate should be fed with pumped breastmilk. If this is not possible, feeding the neonate with pasteurised donor breastmilk or infant formula until breastfeeding can be resumed is recommended.

## Figures and Tables

**Table 1:** Differences between the frequency of signs and symptoms of COVID-19, colds, allergies and flus. Source: Prepared by the authors based on [5].

Sign or Symptom	COVID-19	Cold	Allergies	Flu
Cough	Generally (dry cough)	Generally	Sometimes	Generally
Muscle pain	Generally	Sometimes	Never	Generally
Fatigue	Generally	Sometimes	Sometimes	Generally
Throat pain	Generally	Generally	Rarely	Generally
Blocked or runny nose	Generally	Generally	Generally	Generally
Fever	Generally	Sometimes	Never	Generally—not always
Loss of taste/smell	Generally (initially, often without a blocked nose)	Sometimes (especially with a blocked nose)	Sometimes	Rarely
Shortness of breath/trouble breathing	Generally			Generally
Diarrhoea	Sometimes	Never	Never	Sometimes (more common in children)
Nausea or vomiting	Sometimes	Never	Never	Sometimes (more common in children)
Conjunctivitis	Sometimes		Sometimes	
Sneezing	Rarely	Sometimes	Generally	
Itchy nose, eyes, mouth, or ears	Never		Generally	

**Table 2:** RNA analysis of SARS-CoV-2 in human breastmilk.

Ref.	Year	Type of Study	Sample	Results	Conclusions
[54]	2020	Observational, prospective study	7	Of the seven breastmilk samples collected from infected mothers, all were negative for SARS-CoV-2 by RT-PCR.	Breastmilk was not a source of SARS-CoV-2 transmission. Expressing breastmilk manually, when direct breastfeeding is not possible, appears to be a safe way to feed newborns of mothers who are infected with COVID-19.
[55]	2020	Observational, prospective study	10	Of the ten breastmilk samples collected from infected mothers, all were negative for SARS-CoV-2 by RT-PCR.	Breastmilk was not a source of SARS-CoV-2 transmission. The most important strategies in preventing neonatal SARS-CoV-2 infection are to prevent maternal infection and reduce the possibility of neonatal exposure to the virus.
[56]	2020	Observational study	6	Of the six breastmilk samples collected from infected mothers, all were negative for SARS-CoV-2 by RT-PCR.	SARS-CoV-2 was not found in breastmilk.
[57]	2020	A case report	2	Of the two breastmilk samples collected from infected mothers, all were negative for SARS-CoV-2 by RT-PCR.	Health workers must protect, motivate and encourage breastfeeding. The most likely mother-child transmission is through respiratory droplets.
[58]	2020	A case report	2	Of the 11 breastmilk samples that were collected from two infected mothers (four samples from Mother 1, seven samples from Mother 2), the four samples from Mother 1 were negative and the first four samples from Mother 2 were positive while the last three were negative for SARS-CoV-2 by RT-PCR.	Whether the neonate was infected through breastmilk or other modes of transmission is unknown. Further studies are needed.
[59]	2020	A case report	5	Of the 11 breastmilk samples that were collected from five infected mothers (one sample from Mother 1, two samples from Mother 2, two samples from Mother 3, two samples from Mother 4, one sample from Mother 5), only two samples from Mother 3 were positive for SARS-CoV-2 by RT-PCR.	Conclusions are limited due to the small sample size.
[60]	2020	A case report	1	The neonate was fed breastmilk in the mother’s room and did not become infected.	Encourage breastfeeding and safe room sharing.
[61]	2020	A retrospective case series	22	The neonates were fed breastmilk (20/22) and infant formula (2/22). Nine of the 11 symptomatic mothers were isolated. No infants were infected.	Breastfeed with precautions, after maternal isolation: donor human breastmilk or infant formula until breastfeeding is resumed.
[62]	2020	A case report	1	The breastmilk sample collected from the infected mother was negative for SARS-CoV-2 by RT-PCR. The neonate was fed breastmilk after isolation from the mother.	Breastfeeding after isolation and negative test. Feed the neonate pumped breastmilk during isolation.
[63]	2020	A case report	1	The breastmilk sample collected from the infected mother was positive for SARS-CoV-2 by RT-PCR. Breastfeeding was discontinued after detection of the virus in breastmilk.	Decision of whether to breastfeed should be taken by parents and doctor.
[64]	2020	A case report	1	The breastmilk sample collected from the infected mother was negative for SARS-CoV-2 by RT-PCR. The neonate was breastfed breastmilk pumped from the mother and separated from their mother. The neonate was not infected.	SARS-CoV-2 is rarely transmitted through breastmilk. In addition, there may be an induction of passive immunity from IgG.
[65]	2020	A case report	1	Breastfeeding was discontinued after the mother’s diagnosis and the infant was separated. The neonate was not infected.	Temporarily stop breastfeeding.
[66]	2020	A case report	1	The breastmilk sample collected from the infected mother was negative for SARS-CoV-2 by RT-PCR. The neonate was fed pumped breastmilk directly after isolation from the mother.	Breastfeed cautiously when virus is not found in breastmilk.
[67]	2020	A case report	1	The breastmilk sample collected from the infected mother was negative for SARS-CoV-2 by RT-PCR.	Transmission of the infection from mother to child is very unlikely.
[68]	2020	A case report	1	The breastmilk sample collected from the infected mother was negative for SARS-CoV-2 by RT-PCR. The neonate was breastfed.	Breastfeeding is safe.
[69]	2020	A case report	1	The breastmilk sample collected from the infected mother was positive for SARS-CoV-2 by RT-PCR. Direct breastfeeding was stopped and pumped breastmilk was given when it was confirmed that the infant had been infected with COVID-19.	Breastfeeding should be continued in infected nursing babies. There were no adverse effects. Finding SARS-CoV-2 RNA in the breastmilk sample does not indicate a viable virus.
[70]	2020	A case report	1	Infant formula was given to the neonate and direct breastfeeding was resumed after the baby tested positive.	Encourage breastfeeding and safe room sharing when both mother and infant are infected.
[71]	2020	A case report	1	The breastmilk sample collected from the infected mother was positive for SARS-CoV-2 by RT-PCR. The neonate was breastfed as they were also infected.	More studies are needed to identify transmission routes.
[72]	2020	A case report	1	The breastmilk sample collected from the infected mother was positive for SARS-CoV-2 by RT-PCR. The neonate was fed infant formula.	Breastfeeding can have a protective effect on the infant.
[73]	2020	A case report	1	The breastmilk sample collected from the infected mother was negative for SARS-CoV-2 by RT-PCR. The neonate was breastfed pumped breastmilk from the mother and separated from their mother.	Do not breastfeed when both mother and infant are infected.
[74]	2020	A case series	2	The two breastmilk samples collected from infected mothers were negative for SARS-CoV-2 by RT-PCR. The neonates were fed infant formula. One of the two neonates was separated from their mother.	There is a low risk of vertical transmission through breastmilk. Furthermore, it plays a potentially protective role for passive antibodies.
[75]	2020	Observational, retrospective study	36	Thirty-two of the 36 neonates were breastfed, both directly and pumped. Nine of the 36 neonates were separated from their mother. Thirty-four of 36 neonates tested negative for SARS-CoV-2 and two tested positive.	Support of direct breastfeeding or fed with pumped breastmilk with precautions.
[76]	2020	Prospective, multicentre study	61	Forty-five of 62 neonates were directly breastfed, 13 were directly breastfed and fed infant formula, three were fed infant formula, and one was directly breastfed and fed pumped breastmilk. Eleven of 62 neonates were separated from their mother. No neonate was positive for SARS-CoV-2 at birth and two from 62 were positive at seven and 20 days of life, respectively.	Encourage breastfeeding and room sharing with good conditions when mothers are infected.
[77]	2020	Transversal, retrospective study	45	Thirty-one of 33 neonates were breastfed. Thirty-three of 45 neonates were not separated from the mother. Forty-two of 45 neonates tested negative for SARS-CoV-2.	Encourage breastfeeding and appropriate safe room sharing.
[78]	2020	Collaborative, observational, prospective study	14	Of the 14 breastmilk samples collected from 14 infected mothers, 13 were negative for SARS-CoV-2 by RT-PCR. Eleven of 12 neonates were exclusively breastfed. Neonates were not separated from mothers. Four of 12 neonates tested positive for SARS-CoV-2.	Encourage breastfeeding or feeding with pumped breastmilk (when in isolation) regardless of results with appropriate precautions.
[79]	2020	A case report	One (not infected)	The neonate tested positive for SARS-CoV-2. They were exclusively breastfed and not separated from the mother	More studies are needed to identify transmission routes.

**Table 3:** Analysis of antibodies to SARS-CoV-2 in human breastmilk.

Ref.	Year	Type of Study	Sample	Results	Conclusions
[45]	2021	Prospective cohort group study	131 (31 lactating women)	IgA, IgM and IgG antibodies were found in the 31 breastmilk samples from vaccinated mothers. The second dose of the vaccine produced an increase in SARS-CoV-2-specific IgG, but not in IgA. Immune transfer to neonates was observed through breastmilk.	COVID-19 mRNA vaccines generated robust humoral immunity in lactating women. Immune transfer to neonates occurred through the placenta and breastmilk.
[80]	2020	Ambispective, observational clinical analysis	4	Three breastmilk samples tested positive for SARS-CoV-2 IgM or IgG. Three neonates tested positive for IgG. One neonate tested positive for IgM within 24 h after birth.	Breastfeeding has a low risk of transmitting SARS-CoV-2. Mothers should continue to breastfeed, but take precautions. Babies may benefit from direct acquisition of SARS-CoV-2 antibodies
[81]	2021	Prospective longitudinal study	18	Of the 18 breastmilk samples collected from the 18 infected mothers, all tested negative for SARS-CoV-2 by RT-PCR. Breastmilk contained anti-SARS-CoV-2 IgA and IgG that neutralised the activity of SARS-CoV-2.	Breastmilk produced by infected mothers is a beneficial source of anti-SARS-CoV-2 IgA and IgG and is capable of neutralising the activity of the virus. These results support recommendations to continue breastfeeding during mild to moderate illness.
[82]	2020	A case report	1	The breastmilk sample collected from the infected mother was negative for SARS-CoV-2 by RT-PCR. IgA and IgG antibodies were found in the sample.	IgG and IgA in breastmilk can provide immune protection.
[83]	2021	Prospective cohort group study	84 (504 breastmilk samples)	IgA antibodies were found in 86.1% of the samples and IgG in 97% of the samples. These antibodies showed neutralising effects of the virus.	Robust secretion of SARS-CoV-2 specific IgA and IgG was found in breastmilk after maternal vaccination with virus neutralisation, suggesting a potential protective effect against infection in the infant.

**Table 4:** Studies appraised using the Joanna Briggs Institute critical appraisal checklist for case report.

	Were Patient’s Demographic Characteristics Clearly Described?	Was the Patient’s History Clearly Described and Presented as a Timeline?	Was the Current Clinical Condition of the Patient on Presentation Clearly Described?	Were Diagnostic Tests or Methods and the Results Clearly Described?	Was the Intervention(s) or Treatment Procedure(s) Clearly Described?	Was the Post-Intervention Clinical Condition Clearly Described?	Were Adverse Events (Harms) or Unanticipated Events Identified and Described?	Does the Case Report Provide Takeaway Lessons?	Score Out of 8 (100%)
Lowe et al., 2020 [60]	U	Y	Y	Y	NA	NA	N	Y	4 (50%)
Lang et al., 2020 [62]	U	Y	Y	Y	NA	NA	Y	Y	5 (62.5%)
Bastug et al., 2020 [63]	Y	Y	Y	Y	NA	NA	N	Y	5 (62.5%)
Chu et al., 2020 [64]	Y	Y	Y	Y	NA	NA	N	Y	5 (62.5%)
Feng et al., 2020 [65]	U	Y	Y	Y	NA	NA	N	Y	4 (50%)
Perrone et al., 2020 [66]	U	N	N	Y	NA	NA	N	Y	2 (25%)
Li et al., 2020 [67]	Y	Y	Y	Y	NA	NA	N	Y	5 (62.5%)
Yu et al., 2020 [68]	Y	Y	Y	Y	NA	NA	N	Y	5 (62.5%)
Tam et al., 2021 [69]	N	N	Y	Y	NA	NA	N	Y	3 (37.5%)
Phadke et al., 2020 [70]	Y	Y	Y	Y	NA	NA	N	Y	5 (62.5%)
Kirtsman et al., 2020 [71]	Y	Y	Y	Y	NA	NA	N	Y	5 (62.5%)
Hinojosa el al., 2020 [72]	Y	Y	Y	Y	NA	NA	Y	Y	6 (75%)
Wang et al., 2020 [73]	Y	Y	Y	Y	NA	NA	Y	Y	6 (75%)
Le et al., 2020 [79]	Y	Y	Y	Y	NA	NA	N	Y	5 (62.5%)
Dong et al., 2020 [82]	Y	Y	Y	Y	Y	Y	N	Y	7 (87.5%)

**Table 5:** Studies appraised using the Joanna Briggs Institute critical appraisal checklist for case series.

	Were There Clear Criteria for Inclusion in the Case Series?	Was the Condition Measured in a Standard, Reliable Way for All Participants Included in the Case Series?	Were Valid Methods Used for Identification of the Condition for All Participants Included in the Case Series?	Did the Case Series Have Consecutive Inclusion of Participants?	Did the Case Series Have Complete Inclusion of Participants?	Was There Clear Reporting of the Demographics of the Participants in the Study?	Was There Clear Reporting of Clinical Information of the Participants?	Were the Outcomes or Follow-up Results of Cases Clearly Reported?	Was There Clear Reporting of the Presenting Site(s)/Clinic(s) Demographic Information?	Was Statistical Analysis Appropriate?	Score Out of 10 (100%)
Marín et al., 2020 [54]	Y	Y	Y	Y	N	Y	Y	U	U	NA	6 (60%)
Liu et al., 2020 [55]	Y	Y	Y	Y	Y	Y	Y	Y	Y	Y	10 (100%)
Chen et al., 2020 [56]	Y	Y	Y	Y	N	Y	Y	Y	Y	Y	9 (90%)
Salvatori et al., 2020 [57]	N	Y	Y	U	U	Y	N	Y	U	NA	4 (40%)
Groß et al., 2020 [58]	N	Y	Y	NA	NA	Y	Y	Y	N	NA	5 (50%)
Zhu et al., 2020 [59]	N	Y	Y	NA	NA	Y	Y	Y	N	NA	5 (50%)
Pereira et al., 2020 [61]	Y	Y	Y	Y	U	Y	Y	Y	Y	NA	8 (80%)
Fan et al., 2021 [74]	N	Y	Y	N	N	N	Y	Y	Y	NA	5 (62.5%)
Gao et al., 2020 [80]	N	Y	Y	N	NA	N	Y	Y	Y	Y	6 (60%)

**Table 6:** Studies appraised using the Joanna Briggs Institute critical appraisal checklist for analytical cross-sectional studies.

	Were the Criteria for Inclusion in the Sample Clearly Defined?	Were the Study Subjects and the Setting Described in Detail?	Was the Exposure Measured in a Valid and Reliable Way?	Were Objective, Standard Criteria Used for Measurement of the Condition?	Were Confounding Factors Identified?	Were Strategies to Deal with Confounding Factors Stated?	Were the Outcomes Measured in a Valid and Reliable Way?	Was Appropriate Statistical Analysis Used?	Score Out of 8 (100%)
Gray et al., 2021 [45]	N	N	Y	Y	NA	NA	Y	NA	3 (37.5%)
Elhalik et al., 2020 [75]	Y	Y	NA	Y	NA	NA	Y	Y	5 (62.5%)
Ronchi et al., 2021 [76]	Y	Y	NA	Y	NA	NA	Y	Y	5 (62.5%)
Patil et al., 2020 [77]	Y	Y	NA	Y	NA	NA	Y	Y	5 (62.5%)
Bertino et al., 2020 [78]	Y	Y	NA	Y	NA	NA	Y	Y	5 (62.5%)
Pace et al., 2021 [81]	Y	Y	Y	Y	NA	NA	Y	Y	6 (75%)
Perl et al., 2021 [83]	N	Y	Y	Y	NA	NA	Y	Y	5 (62.5%)
